# PROTAC chemical probes for histone deacetylase enzymes

**DOI:** 10.1039/d3cb00105a

**Published:** 2023-07-27

**Authors:** Urvashi Patel, Joshua P. Smalley, James T. Hodgkinson

**Affiliations:** a Leicester Institute of Structural and Chemical Biology, School of Chemistry, University of Leicester Leicester LE1 7RH UK jthodgkinson@le.ac.uk

## Abstract

Over the past three decades, we have witnessed the progression of small molecule chemical probes designed to inhibit the catalytic active site of histone deacetylase (HDAC) enzymes into FDA approved drugs. However, it is only in the past five years we have witnessed the emergence of proteolysis targeting chimeras (PROTACs) capable of promoting the proteasome mediated degradation of HDACs. This is a field still in its infancy, however given the current progress of PROTACs in clinical trials and the fact that FDA approved HDAC drugs are already in the clinic, there is significant potential in developing PROTACs to target HDACs as therapeutics. Beyond therapeutics, PROTACs also serve important applications as chemical probes to interrogate fundamental biology related to HDACs *via* their unique degradation mode of action. In this review, we highlight some of the key findings to date in the discovery of PROTACs targeting HDACs by HDAC class and HDAC isoenzyme, current gaps in PROTACs to target HDACs and future outlooks.

## Abstract 2

A review on current proteolysis targeting chimeras (PROTACs) as chemical probes for histone deacetylase (HDAC) enzymes.

## Graphical abstract

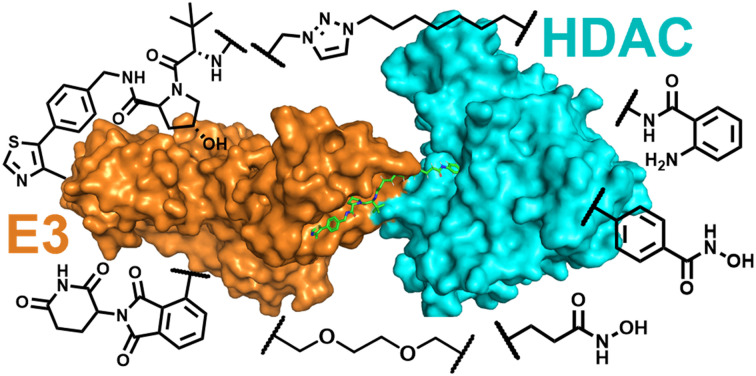

## Introduction to histone deacetylases

Eighteen histone deacetylase (HDAC) enzymes exist in humans, primarily characterised for their catalysis in the hydrolysis of acetyl groups from *N*-ε-acetyl-l-lysine amino acids in histone proteins. The reverse reaction, histone acetylation, can also be carried out by Histone Acetyltransferases (HATs). Histone acetylation and histone deacetylation play important roles in influencing chromatin structure and gene transcription.^1^ For example, histones with non-acetylated lysine residues encompass positive charges and are tightly associated with negatively charged DNA, termed heterochromatin.^2^ Alternatively, histones with acetylated lysine residues, with no charge on the lysine residue, are less tightly associated with DNA and are less compact in histone DNA packing, termed euchromatin.^2^ Additionally, the acetylated lysine residues on histones also serve as important recruitment sites for bromodomain-containing proteins that can further recruit HATs or chromatin remodelers capable of altering DNA accessibility to transcription factors.^3^ Hence, the fine balance between HDAC and HAT activities in the cell plays an important role in the regulation of gene expression, and such post-translational modifications are considered important epigenetic markers.^4^

Despite their role in histone deacetylation, the name Histone Deacetylase can perhaps be deceptive as the substrate specificity of HDACs can expand beyond histone proteins to non-histones such as p53, signal transducer and activator of transcription 3 (STAT3), the androgen receptor (AR) and α-tubulin as a few select examples.^5–8^ p53, STAT3 and AR are important transcription factors whereby their overexpression or mutation can be associated with many cancers.^9–11^ While α-tubulin, another important cancer drug target, is a central component of the cytoskeleton and important for cell division.^12^

Higher eukaryotic organisms typically contain more HDAC isoforms, the common fruit fly only encodes 5 HDAC genes in comparison to the 18 HDACs in humans.^13^ Of the eighteen HDAC enzymes in humans, eleven perform catalysis utilising a divalent Zn^2+^ ion in the HDAC active site, while the remaining seven are NAD^+^-dependent and termed Sirtuins (SIRTs).^14^ HDACs can be further subdivided into classes based on their sequence similarity to yeast HDACs Hda1 and Rpd3. The eleven-zinc dependent HDACs encompass class I (HDAC1, HDAC2, HDAC3 and HDAC8), class II (HDAC4 – HDAC7, HDAC9 and HDAC10) and class IV (HDAC11), and the seven SIRTs encompass class III (SIRT1–SIRT7). Class II can be further subdivided into class IIa (HDAC4, HDAC5, HDAC7, and HDAC9) and class IIb (HDAC6 and HDAC10). These isoforms differ in their sub-cellular locations, catalytic activities and substrate specificities. For example, HDAC1, HDAC2 and HDAC3 are predominantly localised in the nucleus while the majority of other zinc-dependent HDACs can be found in either the nucleus or cytoplasm.^15^

## Small molecule HDAC inhibitors

**Fig. 1:**
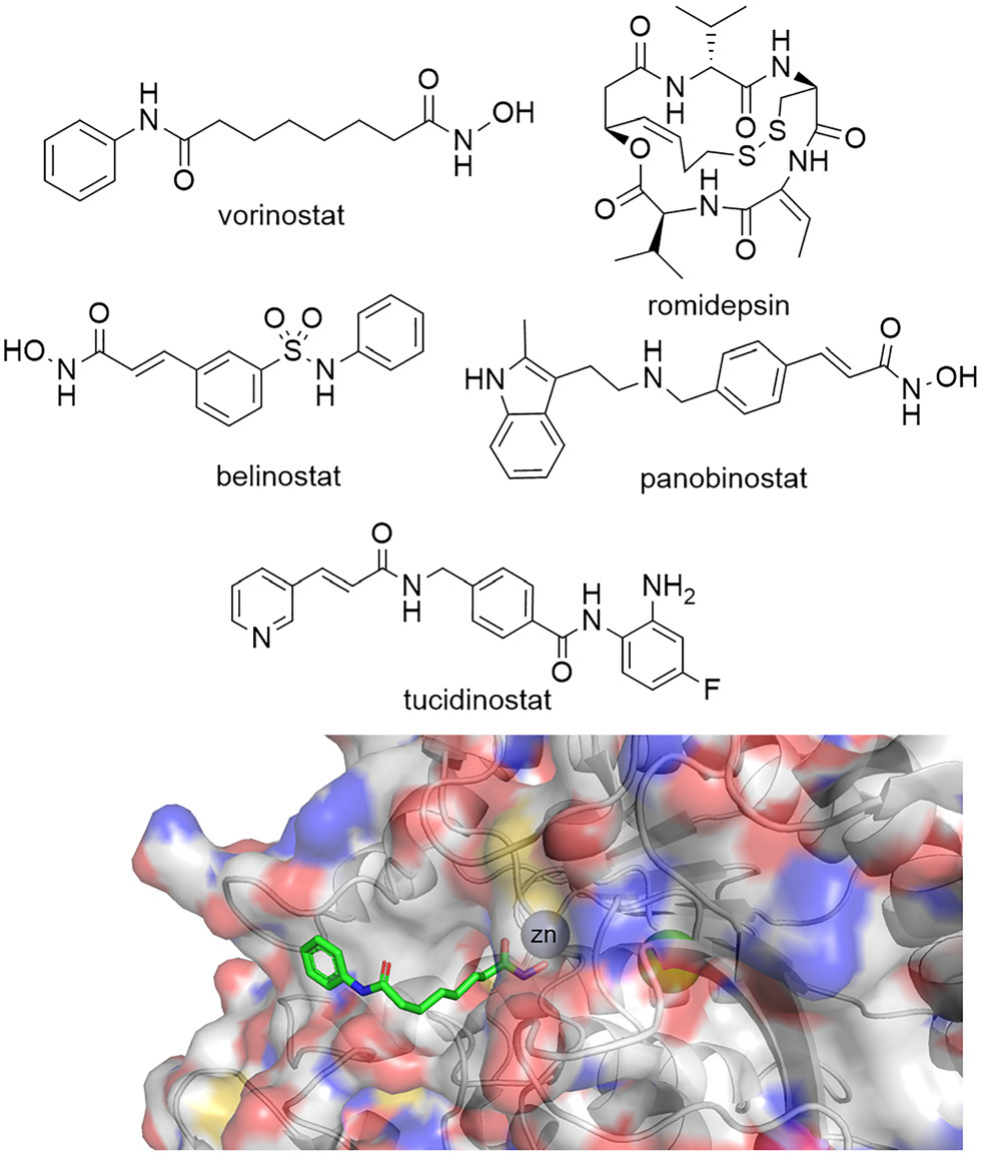
Top: Vorinostat, romidepsin, belinostat and panobinostat are drugs that inhibit the HDAC catalytic active site and are FDA approved. Tucidinostat has been approved for in-patient use in China. Bottom: Crystal structure of HDAC2 with vorinostat bound *via* the hydroxamic acid functional group to Zn^2+^ in the HDAC catalytic active site PDB: 4LXZ.^95^

Given the importance of HDACs in gene transcription it is perhaps no surprise that a number of HDAC inhibitors have gained FDA approval (Fig. 1). Vorinostat, belinostat and romidepsin have been approved for the treatment of T-cell lymphomas, while panobinostat has been approved for multiple myeloma. Tucidinostat, while not FDA approved, has been approved in China for the treatment of peripheral T-cell lymphoma. However, given vorinostat gained FDA approval in 2006 the peril in bringing new HDAC targeting drugs to the clinic has perhaps slightly overshadowed the excitement surrounding their potential as new drugs.^16^ In comparison, since the approval of the first rationally designed kinase inhibitor imatinib in 2001, over 74 small molecule inhibitors targeting kinases gained FDA approved by the end of 2021.^17^ One hurdle to the potential progression of new HDAC inhibitors is perhaps the toxicities that have been associated with current FDA-approved HDAC drugs. Side effects experienced by patients taking vorinostat include fatigue, gastrointestinal upset, thrombocytopenia and QT interval prolongation,^18^ more akin to a non-selective cytotoxic agent than a targeted cancer therapeutic. Many have hypothesised that these side effects are linked to the pan-HDAC inhibition of all eleven zinc-dependent HDAC enzymes by inhibitors such as vorinostat.^16,19–22^ It could be envisaged the global change in the acetylome caused by pan-HDAC inhibition could be leading to these undesired side effects. However, it is also important to note that the hydroxamic acid functional group, incorporated in three of four FDA approved HDAC targeting drugs, have also been associated with mutagenicity.^23^

Despite this there is still hope and enthusiasm that the discovery of isoform selective HDAC inhibitors will lead to enhanced therapeutic efficacies with reduced side effects for specific diseases related to certain HDAC isoforms.^16,19–22^ The literature on small molecule HDAC inhibitors is vast and beyond the scope of this review. We recommend the review by Ho *et al.* for a comprehensive account of HDAC inhibitor design and development over the past thirty years.^24^ At the time of writing this review 135 clinical trials have been reported as active or recruiting in clinicaltrials.gov related to the search term ‘Histone Deacetylase’.^25^ Many of these trials are combination therapies with FDA approved HDAC inhibitors, but also contain new HDAC inhibitors, and hybrid dual functionalised HDAC inhibitors designed to target HDACs and other protein drug targets including kinases.^26^ Intriguingly, current HDAC inhibitors targeting a singular HDAC isoform in clinical trials are mainly represented by HDAC6.

Beyond isoform selectivity, HDAC1, HDAC2 and HDAC3 exist *in vivo* in seven differing corepressor complexes adding an additional layer of complexity.^27^ The differing corepressor complexes each have distinct physiological roles in the cell, and hence targeting a HDAC containing corepressor complex with selectivity may also be essential to discovering new HDAC therapeutics with greater therapeutic efficacies and reduced side effects.^28^ It will be truly interesting to see how the future of Proteolysis Targeting Chimeras (PROTACs) can contribute to this area.

## PROTACs as chemical probes

Small molecule chemical probes that exhibit high affinity and selectivity for their intended target protein serve important applications in addressing fundamental biological questions related to the target protein.^29–31^ Chemical probes have also proved important for validating the role of specific proteins in disease and their potential as drug targets.^32,33^ Most chemical probes are rationally designed to inhibit protein function. However, the discovery of cell permeable molecules, not only capable of protein inhibition, but also degradation of the target protein has created a new area of research in chemical probes.^34–36^ Perhaps the most well-known examples of this being proteolysis targeting chimeras (PROTACs). However, given the success of this strategy other protein degradation strategies such as molecular glues, lysosome-targeting chimeras (LyTACs), autophagosome-tethering compounds (ATTECs) and antibody-based PROTACs (AbTACs) are also being actively explored.^37–39^ To date, the majority of protein degradation strategies targeting HDACs has been focused on the design of PROTACs which will also be the focus of this review, however Toriki *et al.* recently reported molecular glues targeting HDAC1 and HDAC3 for degradation.^40^

**Fig. 2:**
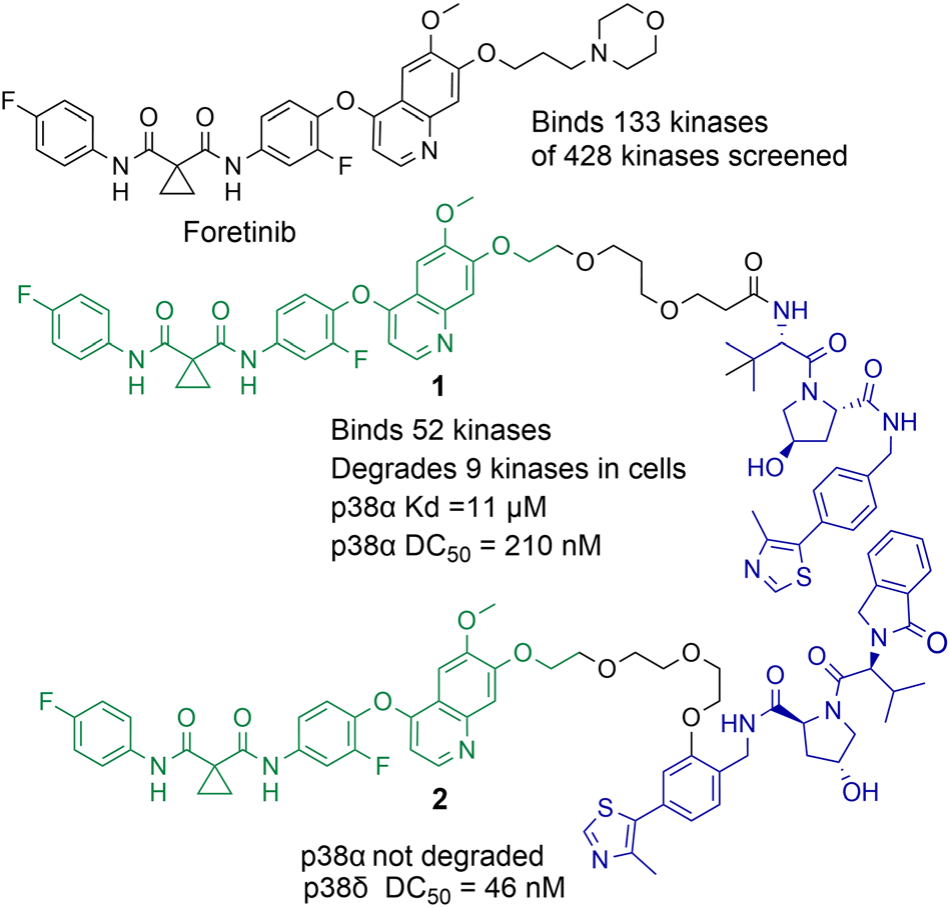
Example of how the pan-kinase inhibitor foretinib can be transformed into a selective kinase protein degrader.^44,45^

PROTACs designed to degrade their target protein typically contain three components: a ligand to engage the protein of interest (POI), a ligand to initiate protein degradation (most commonly an E3-ligand), and a linker covalently bonding these two ligands. PROTACs result in poly-ubiquitination of the lysine amino acids on the POI resulting in eventual degradation by the proteasome. This transfer of ubiquitin to the POI requires the formation of a protein–protein interaction between the POI and E3-ligase mediated by the PROTAC,^41^ the formation of a stabilised and cooperative ternary complex is often highlighted as important for degradation.^42^ This unique mechanism of action can lead to significantly enhanced protein selectivity. For example, the bromodomain inhibitor JQ1 is a pan-BET inhibitor, however when JQ1 is incorporated into a PROTAC, this results in the selective degradation of BRD4 over BRD2 and BRD3.^43^ Foretinib, a pan-kinase inhibitor, was found to bind 133 kinases of the 428 kinases screened by Bondeson *et al.*,^44^ however when functionalised into PROTAC 1 (utilising the Von Hippel-Lindau E3-ligand) it retained binding to just 52 of the 133 kinases (Fig. 2). In proteomic studies, involving 1 a total of 36 proteins were found to be degraded. However, only 9 of the 54 kinases known to bind foretinib (investigated in the proteomics study) were degraded by 1. Another remarkable finding in the study was that tighter PROTAC-kinase binding affinity did not necessarily correlate with more effective kinase degradation, for example, 1 exhibited a *K*_d_ value of 11 μM for p38α but exhibited a DC_50_ (concentration that causes 50% protein degradation) of 210 nM for p38α. The stability of the ternary complex formed between 1, p38α and the VHL ligase complex was highlighted as more important for degradation than the binding affinity between p38α and 1. Further studies revealed that modifying the connectivity of the VHL ligand to the linker in PROTAC 2, while maintaining the same foretinib POI binding ligand, can modify the selectivity of degradation for the p38δ isoform over previously degraded p38α.^45^ These are just a few examples of how PROTAC-mediated degradation can alter protein selectivity, and there are many recent and comprehensive reviews available on PROTACs.^46–48^

Given such remarkable findings in modifying protein selectivity by utilising PROTAC mediated degradation over enzyme/receptor inhibition it is no surprise researchers wishing to modulate HDAC activity have been drawn to the PROTAC field. The first PROTAC targeting a Histone Deacetylase enzyme was NAD^+^-dependent SIRT2 published online in 2017.^49^ The first zinc-dependent HDAC targeting PROTAC was reported for HDAC6 in 2018.^50^ It is important to note PROTACs also have promise beyond chemical probes with approximately 20 PROTACs progressing to or already in clinical trials.^46^ The most prevalent protein targets for these PROTACs being AR, estrogen receptor (ER), Bruton's tyrosine kinase (BTK) and interleukin-1 receptor-associated kinase 4 (IRAK4). To date, as of writing, no PROTACs targeting HDACs are in clinical trials.

## PROTACs targeting Class I HDACs

Class I HDACs encompass HDAC1, HDAC2, HDAC3 and HDAC8. HDAC1, HDAC2 and HDAC3 are predominantly localised in the nucleus of the cell, while HDAC8 can be found in both the nucleus and cytoplasm.^28^ HDAC1 and HDAC2 share 83% amino acid sequence identity and exist *in vivo* interchangeably as components of six corepressor complexes (CoREST, NuRD, Sin3, MiDAC, RERE and MIER), while HDAC3 exists in the SMRT/NCoR complex.^28^ HDAC8, unlike HDAC1-3, is not known to exist in corepressor complexes.

Of the HDAC family, HDAC1–HDAC3 are attractive drug targets given that they are nucleus-localised and hence likely the most prominent HDACs in regulating histone deacetylation and gene transcription. The selective targeting of HDAC1 and HDAC2 has been highlighted as a potential strategy for treating B-cell acute lymphoblastic leukaemia (B-ALL),^51^ while HDAC3 has recently been shown to have a critical role in tumour development in Kras-mutant non-small cell lung cancer.^52^ HDAC8 has been found to be overexpressed in a number of cancers.^53^ To date, a number of PROTACs have been reported capable of degrading class I HDACs.^54–68^

## PROTACs targeting HDAC1 and HDAC2

Although PROTACs have been reported capable of degrading HDAC1 and HDAC2 achieving the selective degradation of these isoforms has proved challenging. Xiong *et al.* carried out a proteomics study investigating HDAC degradation in the presence of 48 PROTACs that varied in HDAC ligand, linker length and E3 ligand.^56^ In this study HDAC1, HDAC2 and HDAC9 were the least frequently identified HDAC isoforms to undergo proteasome mediated degradation of 9 of the 11 zinc-dependent HDACs on PROTAC treatment (HDAC10 and HDAC11 are in low abundance in the KELLY cell line used in the study). On the other side of the spectrum, HDAC6, HDAC8 and HDAC3 were the most frequently identified HDAC isoforms to undergo proteasome mediated degradation with the 48 PROTACs used in the study. This seems to be the challenge in generating HDAC1 and HDAC2 selective PROTACs, separating HDAC1 and HDAC2 degradation from other isoform degradation such as HDAC3 and HDAC6 degradation, the latter of which seem more susceptible to PROTAC mediated degradation, and there are other examples of this beyond the study by Xiong *et al.*

**Chart 1:**
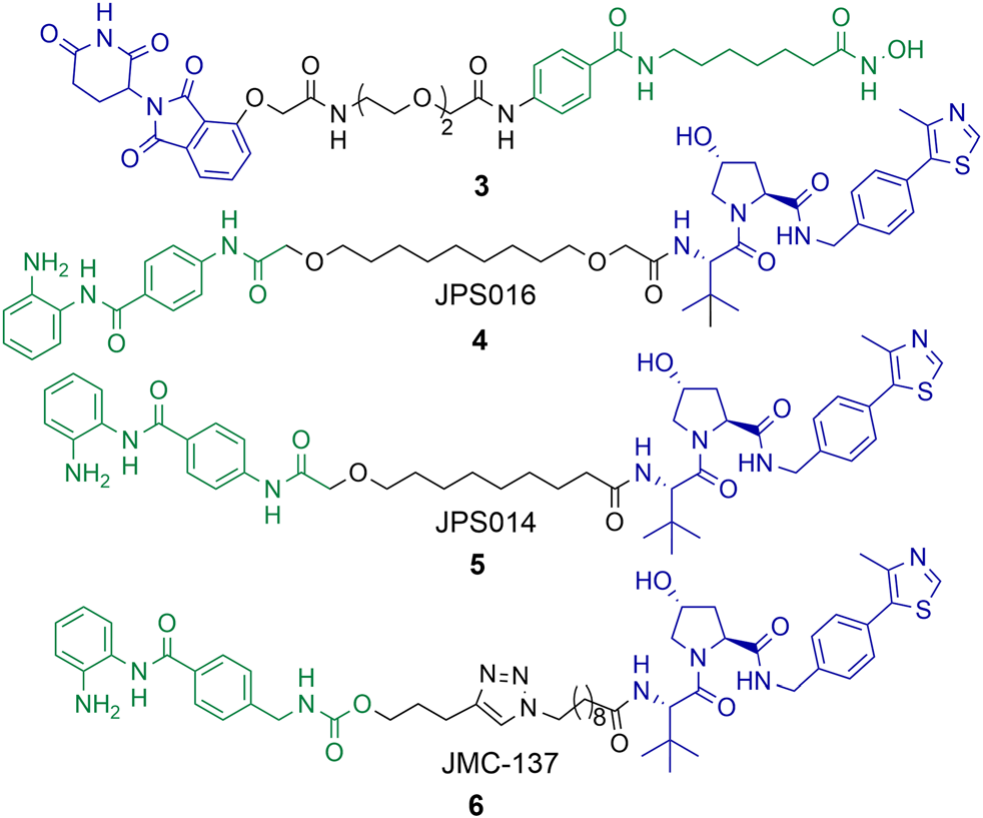
PROTAC 3 degrades HDAC1 at a concentration of 10 μM, but also effectively degrades HDAC6 at 1 μM in HL-60 cells.^57^4 and 5 degrade HDAC1 with DC_50_ values of 0.55 μM and 0.91 μM respectively in HCT-116 cells and degrade HDAC2,^58^5 exhibits a DC_50_ value of 4.19 μM for HDAC2. HDAC3 is degraded with 4 and 5 with DC_50_ values of 0.53 μM and 0.64 μM respectively, however a hook effect is observed for HDAC3 at concentrations >1 μM which is not observed for HDAC1 and HDAC2. 6 degrades HDAC1 with a DC_50_ value of 2.8 μM and also degrades HDAC2 and HDAC3, 6 exhibits a similar hook effect with HDAC3 at concentrations >2.5 μM.^59^

Using a novel hydroxamic acid immobilised resin (HAIR) approach Sinatra *et al.* were able to synthesise PROTAC 3, which was capable of degrading HDAC1 at a concentration of 10 μM in the acute myeloid leukaemia HL-60 cell line (Chart 1).^57^ However, HDAC6 was also degraded with 3 and even more effectively degraded than HDAC1 at lower concentrations. As an alternative to PROTACs based on hydroxamic acids to target HDACs we have been investigating HDAC1-3 selective benzamides as the HDAC binding ligand in PROTACs.^54,58^ PROTACs 4 and 5 were identified as degraders of HDAC1 and HDAC2 in the colorectal carcinoma HCT116 cell line.

PROTAC 4 exhibited a DC_50_ value of 0.55 μM for HDAC1 and still degraded HDAC2 at low micromolar concentrations, while 5 exhibited DC_50_ values of 0.91 μM and 4.19 μM respectively for HDAC1 and HDAC2.^58^ These PROTACs are also effective degraders of HDAC3 (DC_50_ values of 0.53 μM and 0.64 μM), however we noticed a significant observation in that these PROTACs exhibit a hook effect for HDAC3. At concentrations greater than 1 μM HDAC3 abundance starts to increase rather than decrease, while reduced HDAC1 and HDAC2 abundance was still maintained at concentrations greater than 1 μM. This effect was also time dependent and at 36 hours HDAC3 abundance started to reduce again with 4 meaning caution must be taken not only with the PROTAC concentration used but also with time-dependent degradation. PROTACs 4 and 5 were capable of compromising cell viability in HCT116 cells with single-digit micromolar IC_50_ values, however they were only marginally enhanced in potency compared to the benzamide class I HDAC inhibitor CI-994. In a separate study, we investigated utilising the class I HDAC inhibitor entinsotat as inspiration for PROTAC 6.^59^ PROTAC 6 encompassing the triazole and carbamate moiety was also a degrader of HDAC1, HDAC2 and HDAC3 in HCT116 cells, but was reduced in degradation potency (HDAC1 DC_50_ = 2.8 μM) compared to 4 and 5. PROTAC 6 also exhibited a similar hook effect for HDAC3 although at higher concentrations, above 2.5 μM.

A real feat of the PROTAC technology would to be selectively degrade HDAC1 over HDAC2 or *vice versa* given the high amino acid sequence similarity between HDAC1 and HDAC2. With PROTACs 4 and 5 the dose response curves for HDAC1 and HDAC2 degradation mirror one another, however we have consistently observed greater HDAC1 degradation levels over HDAC2 degradation,^54,58,59^ at least suggesting this may be viable. Regards targeting HDAC1 and HDAC2 for degradation, there are PROTACs reported that degrade HDAC1 and HDAC2, however not with complete selectivity and work still needs to be done to fine tune HDAC1 and HDAC2 degradation over the degradation of other HDAC isoforms.

## PROTACs targeting HDAC3

**Chart 2:**
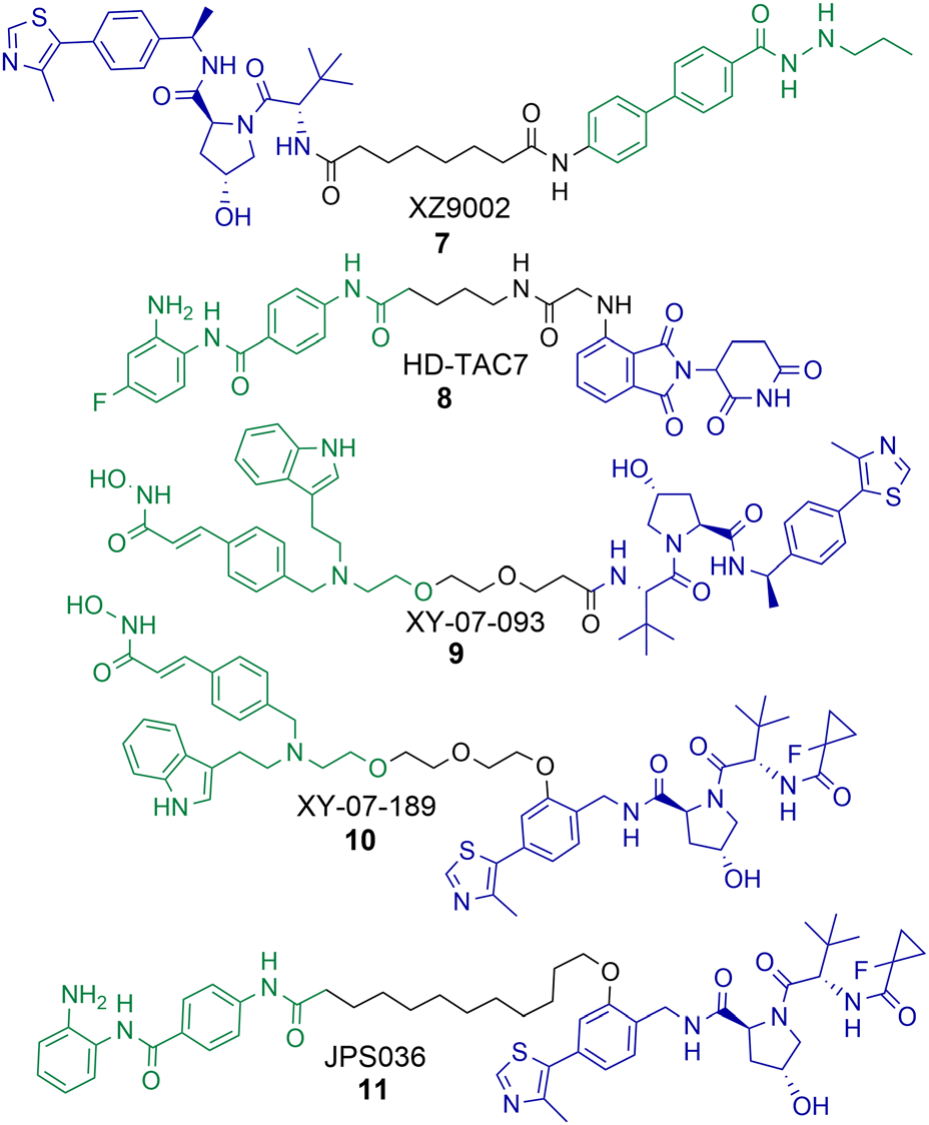
PROTACs reported for the selective degradation of HDAC3. 7 HDAC3 DC_50_ = 42 nM and 7 exhibits selectivity over HDAC1, HDAC2 and HDAC6 in MDA-MB-468 cells.^60^8 HDAC3 DC_50_ = 0.32 μM in RAW 264.7 macrophages.^61^9 and 10 were identified in a proteomics study by Xiong *et al.* with selectivity for HDAC3 degradation in KELLY cells, however this HDAC3 selectivity was completely compromised in MM.1S cells.^56^11 HDAC3 DC_50_ = 0.44 μM and exhibits selectivity over HDAC1 and HDAC2 in HCT116 cells.^58^

There have been a number of PROTACs reported that degrade HDAC3 with selectivity over other HDAC isoforms.^56,58,60,61^ Using a hydrazide pan HDAC1-3 inhibitor, an uncommon HDAC ligand, Xiao *et al.* were able to synthesise and identify a HDAC3 selective degrader, 7, incorporating an alkyl linker and a VHL ligand in the PROTAC (Chart 2).^60^ PROTAC 7 exhibited an impressive DC_50_ value of 42 nM in breast cancer MDA-MB-468 cells with no significant changes in HDAC1, HDAC2 or HDAC6 abundance. Intriguingly, 7 was not as potent at inducing hyperacetylation and compromising cell viability as the pan-HDAC1-3 inhibitor from which it was originally derived in three different breast cancer cell lines. However, 7 was more effective at compromising cell viability than its negative control analogue incapable of engaging VHL and promoting HDAC3 degradation, suggesting that HDAC3 degradation over HDAC3 inhibition is more effective in compromising cell viability in the cell lines tested.

Cao *et al.* reported a benzamide based cereblon recruiting PROTAC, 8, that degrades HDAC3 with a DC_50_ value of 0.32 μM in RAW 264.7 macrophages.^61^ This is a rarer example of a cereblon recruiting HDAC3 PROTAC reported in the literature. The authors wanted to investigate the effects of 8 on inflammation pathways, however in comparison to the pan-HDAC1-3 inhibitors CI-994 and entinsotat, 8 did not significantly alter gene expression of IL-10, IL-6, TNFα and iNOS. The authors highlight that the use of pomalidomide as the E3-ligand in 8 may be responsible for this.

In the proteomics study carried out by Xiong *et al.* PROTACs 9 and 10 were identified as selective degraders of HDAC3 in KELLY cells.^56^ A remarkable observation was that when 9 and 10 were evaluated in MM.1S cells this HDAC3 selective degradation was completely abolished. In MM.1S cells at least 6 other HDAC isoforms were degraded in the presence of 9 and 5 other HDAC isoforms in the presence of 10. This highlights how the cell line the PROTAC is tested in can be crucial to the HDAC isoform degradation observed. Xiong *et al.* also observed that VHL recruiting PROTACs were more effective in promoting the degradation of HDAC3 compared to PROTACs recruiting cereblon and IAP E3-ligases, highlighting that for designing PROTACs to selectively degrade HDAC3 the VHL ligand may be the E3 ligand of choice.

In optimisation studies of PROTACs targeting HDAC1-3 for degradation we modified the attachment of the VHL E3-ligand to the linker in PROTAC 11.^58^ Attaching VHL to the linker *via* an ether bond on the phenyl ring of VHL in 11, rather than the amide bond used in PROTACs 4 and 5, resulted in abolishment of the hook effect that we previously observed for HDAC3. Additionally, 11 exhibited HDAC3 degradation with a DC_50_ value of 0.44 μM in HCT116 cells, comparatively at 0.5 μM no degradation of HDAC2 was observed and only approx. 10% degradation of HDAC1. Similar to the results observed by Xiao *et al.* with 7, PROTAC 11 did not result in increased Histone3 Lysine56 acetylation (H3K56ac) compared to 4 and 5 that also degrade HDAC1 and HDAC2. 11 also did not compromise cell viability in HCT116 cells to the same levels as PROTACs 4 and 5 and the pan-HDAC1-3 inhibitor CI-994.

The selective degradation of HDAC3 by PROTACs seems to be achievable. PROTACs that recruit the VHL E3 ligase are more likely to promote the degradation of HDAC3. However, it is also important to note that the choice of cell line can have a crucial effect on the HDAC isoform degradation outcome.

## PROTAC effects on HDAC1-3 containing corepressor complexes

Very few studies have investigated the effects of HDAC targeting PROTACs on the corepressor complexes that HDAC1, HDAC2 and HDAC3 exist *in vivo*.^56,62^ One of few studies was carried out by Xiong *et al.*, investigating the effects of 48 PROTACs on HDAC1-3 containing corepressor complexes by proteomics.^55^ Reduced abundance of GPS2, NCoR1 and NCoR2, components of the HDAC3-SMRT/NCoR complex, coincided with PROTACs capable of degrading HDAC3, including PROTACs 9 and 10. The reduced abundance of these HDAC3-SMRT/NCoR corepressor complex components was much more prominent with PROTACs recruiting the VHL E3 ligase compared to PROTACs recruiting the cereblon E3 ligase (HDAC ligand dacinostat). Intriguingly, MIER1–3, components of HDAC1/2 containing corepressor complexes, were also reduced in abundance with certain PROTACs, including 9 and 10, despite no observed significant reduction in HDAC1 and HDAC2 abundance. Three PROTACs recruiting the IAP E3 ligase were capable of reducing the abundance of GSE1 and HMG20B components of the CoREST complex. The authors hypothesised that such PROTACs can engage HDAC1 and HDAC2 in these corepressor complexes but only result in the degradation of these specific complex components and not the degradation of HDAC1 and HDAC2 themselves.

We performed RNA-seq experiments with the HDAC1–3 inhibitor CI-994 and seven PROTACs capable of degrading HDAC1–3 with different degradation profiles side-by-side (each PROTAC, CI-994 and controls screened in three independent biological replicates).^62^ We compared the gene expression profile of multiple components of all seven HDAC1–3 containing corepressor complexes. Although not considered differentially expressed genes, genes of the HDAC3-SMRT/NCoR complex TBL1RX1, TBL1X and NCoR1 were exclusively only upregulated in the presence of the HDAC3 selective PROTAC 11, highlighting again, similar to the results by Xiong *et al.*, that this corepressor complex does seem to be affected as whole by PROTAC-mediated degradation.

More efforts are needed to investigate the effects of PROTAC-mediated degradation on corepressor complexes. This is a real advantage that the PROTAC-mediated degradation of HDACs could offer over HDAC inhibitors. However, discovering such PROTACs for HDAC1/2 complexes is non-trivial as HDAC1 and HDAC2 exist in six different corepressor complexes interchangeably, with each individual corepressor complex encompassing multiple protein partners. Additionally, the selective degradation of one of the six HDAC1/2 corepressor complexes may have minimal effects on HDAC1 or HDAC2 abundance overall, and such PROTACs could easily be overlooked.

## PROTACs targeting HDAC8

**Chart 3:**
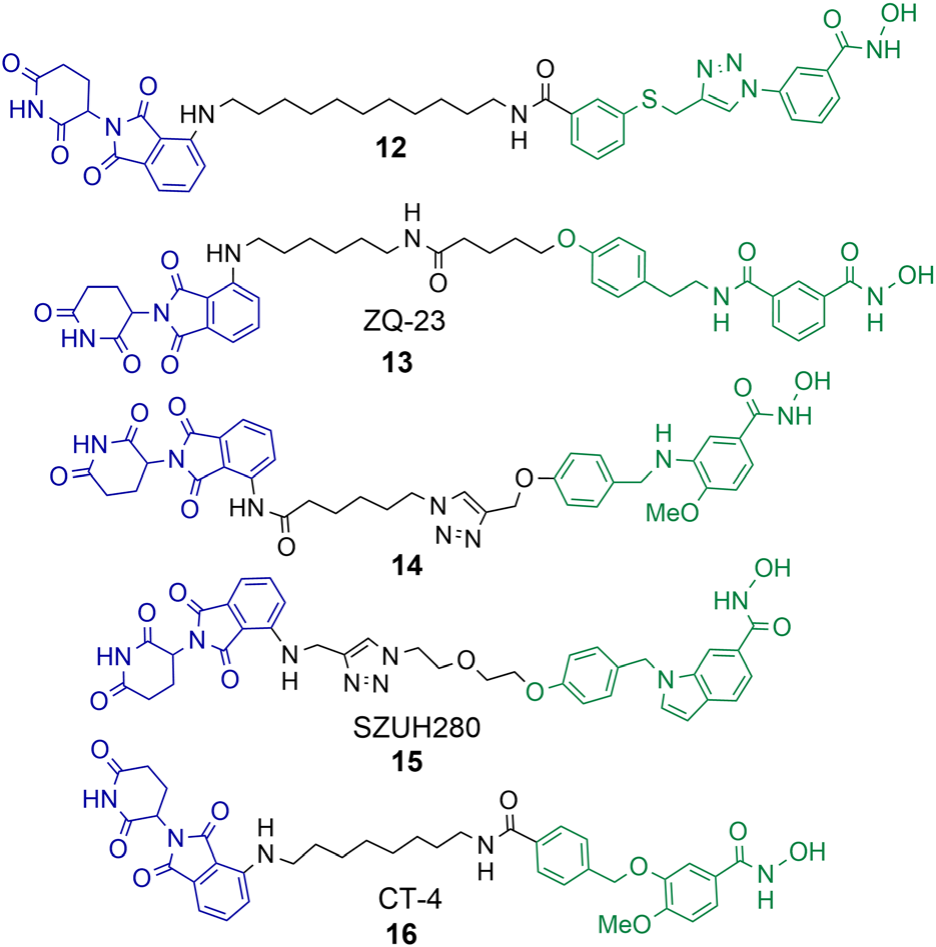
PROTACs reported for the selective degradation of HDAC8. 12 HDAC8 DC_50_ = 0.7 μM in jurkat cells.^64^13 HDAC8 DC_50_ = 147 nM and HDAC6 DC_50_ = 4.95 μM in HCT-116 cells.^65^14 degrades HDAC8 with a *D*_max_ value of 70% at 10 μM in SK-N-BE(2)-C neuroblastoma cells.^66^15 HDAC8 DC_50_ = 0.58 μM in A549 cells.^67^16 HDAC8 DC_50_ = 1.8 nM and HDAC6 DC_50_ value = 38 nM in MDA-MB-231 cells.^68^16 HDAC8 DC_50_ = 4.7 nM and HDAC6 DC_50_ = 78.5 nM in jurkat cells.

A number of studies have now been reported on the PROTAC mediated degradation of HDAC8.^63–68^ Again, this perhaps reflects the observation in the proteomic study by Xiong *et al.* that HDAC8 is one of the HDAC isoforms more prone to PROTAC mediated degradation along with HDAC3 and HDAC6. Utilising a previously developed selective HDAC8 inhibitor Chotitumnavee *et al.* were able to synthesise a selective PROTAC degrader of HDAC8 (Chart 3).^64^ PROTAC 12 degraded HDAC8 in jurkat cells with a DC_50_ value of 0.7 μM, the attachment position of the linker to the HDAC8 engaging ligand was crucial to promote degradation. HDAC1, HDAC2 and HDAC6 were not degraded in the presence of 12. PROTAC 12 outperformed the inhibitor from which it was originally derived with 10-fold enhanced potency in compromising cell viability in jurkat cells. This was an interesting finding given that the parent inhibitor was more potent at HDAC8 inhibition (IC_50_ = 0.05 μM) in comparison to HDAC8 inhibition and degradation by 12 (IC_50_ = 0.37 μM, DC_50_ = 0.7 μM). The authors noted PROTAC 12 also reduced levels of IKZF1 a known neo-substrate for the cereblon E3 ligase, however the E3-ligand pomalidomide alone did not show comparable potency in compromising cell viability. This perhaps suggests that the degradation of HDAC8 by PROTACs may be more effective than the selective inhibition of HDAC8 in compromising cell viability.

Sun *et al.* utilised a dual HDAC6-HDAC8 inhibitor for PROTAC design.^65^ Intriguingly, although the inhibitor from which PROTAC 13 was designed, is a more potent HDAC6 inhibitor than HDAC8 inhibitor, 13 was capable of approx. 30-fold more potent HDAC8 degradation over HDAC6 degradation in HCT-116 cells. 13 exhibited DC_50_ values of 147 nM and 4.95 μM respectively for HDAC6 and HDAC8. The authors suggest that 13 is more effective at forming a ternary complex with HDAC8 over HDAC6. No degradation of HDAC1 or HDAC3 was observed in the presence of 13. Acetylation of SMC-3 a known substrate for HDAC8 was also observed. Degradation with 13 was time-dependent with degradation initiated after 2 hours, reaching maximum HDAC8 degradation levels at 10 hours, and HDAC8 levels started to rise again at 24 hours. No cell viability studies were reported with 13.

Darwish *et al.* synthesised a series of PROTACs designed to selectively target HDAC8 for degradation in neuroblastoma cells, utilising a selective HDAC8 inhibitor as the basis for PROTAC design.^66^ PROTAC 14 was capable of dose-dependent HDAC8 degradation with a *D*_max_ value (maximum percentage degradation) of 70% for HDAC8 at 10 μM and no degradation of HDAC1 or HDAC6 was observed. Degradation was again also time-dependent, with the maximum degradation of HDAC8 exhibited at 6 hours and recovery of HDAC8 to basal levels noted after 48 hours. Comparative VHL recruiting analogues of 14 exhibited no significant HDAC8 degradation. PROTAC 14 was also capable of increasing the acetylation of SMC-3 and exhibited anti-neuroblastoma activity.

Huang *et al.* utilised an indole scaffold, again a selective inhibitor of HDAC8, for the development of PROTACs to target HDAC8.^67^ Unique to this study VHL based PROTACs were capable of effectively degrading HDAC8, however they were again outperformed by cereblon recruiting PROTACs. PROTAC 15 exhibited a DC_50_ of 0.58 μM for HDAC8 in A549 cells with a *D*_max_ value greater than 95%, and maximum HDAC8 degradation was observed at 18 hours. Again, similar to the results observed by Chotitumnavee *et al.* PROTACs outperformed the selective HDAC8 inhibitors from which they were originally derived in compromising cell viability in A549 cells. PROTAC 15 was also capable of long duration tumour regression in an A549 tumour mouse model.

Zhao *et al.* reported a highly potent HDAC8 targeting PROTAC with a single digit nanomolar DC_50_ value of 1.8 nM and *D*_max_ value of 97% in MDA-MB-231 breast cancer cell line.^68^16 was also a degrader of HDAC6 but with a higher DC_50_ value of 38 nM. Rapid HDAC8 degradation was observed at 4 hours with 16, and HDAC8 abundance was reduced up to 48 hours after treatment. When 16 was investigated in jurkat cells the DC_50_ value modestly increased to 4.7 nM for HDAC8 and 78.5 nM for HDAC6 highlighting a modest drop in potency and HDAC8 selectivity in a different cell line. Although 16 only exhibited weak anti-proliferative activity in MDA-MB-231 cells it still outperformed the selective HDAC8 inhibitor from which it was derived and also inhibited migration of MDA-MB-231 cells whereby the selective HDAC8 inhibitor did not. In cell viability assays, and assays investigating apoptosis, 16 was more effective than its parent selective HDAC8 inhibitor. However, at the effective micromolar concentrations needed in these assays concurrent HDAC6 degradation with HDAC8 degradation may not necessarily be ruled out.

## PROTACs targeting Class IIa HDACs

Class IIa HDACs encompass HDAC4, HDAC5, HDAC7 and HDAC9. These HDACs can be found in both the nucleus and cytoplasm and they exhibit significantly reduced deacetylase enzymatic activity on histone proteins compared to class I HDACs.^69^ Li *et al.* reported that the treatment of HEK293 cells with the proteasome inhibitor MG132 results in an increased abundance of HDAC4, HDAC5, and HDAC7, with HDAC7 levels even further elevated again comparatively to HDAC4 and HDAC5.^70^ This suggests these HDAC enzymes are prone to proteasome mediated degradation in cells in their own right. Again, this finding correlates well with the proteomic study by Xiong *et al.*,^56^ after HDAC6, HDAC3 and HDAC8, the isoforms HDAC7, HDAC5 and HDAC4 respectively were the most frequently identified to be in reduced abundance on PROTAC treatment, with no PROTACs reported to reduce the abundance of HDAC9. In terms of gaining isoform selective degradation by PROTAC treatment within the class IIa enzymes only HDAC4 selective PROTACs have been reported to date.

## PROTACs targeting HDAC4

**Chart 4:**
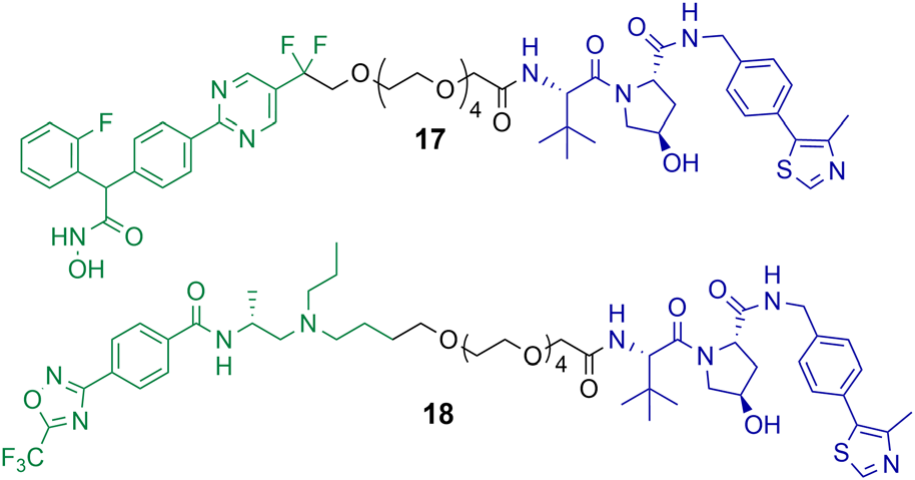
PROTACs reported for the selective degradation of HDAC4. PROTAC 17 DC_50_ = 37 nM and *D*_max_ value of 83% in Jurkat E6-1 cells.^71^ PROTAC 18 DC_50_ = 4 nM and *D*_max_ value of 78% in Jurkat E6-1 cells.^71^ PROTAC 17 exhibits a hook effect with HDAC4 at 10 μM in Jurkat E6-1 cells.^71^

Macabuag *et al.* reported the first HDAC4 selective PROTACs to investigate the role of HDAC4 in Huntington's Disease (Chart 4).^71^ They developed two sets of isoform-selective PROTACs. The first series incorporated a hydroxamic acid-based inhibitor, analogous to the CNS penetrant class IIa HDAC inhibitor developed by Luckhurst *et al.*,^72^ which was appended *via* a PEG linker to a VHL E3 ligase ligand, with three different lengths investigated. The second series of PROTACs were based on a trifluoromethyl oxadiazole (TFMO) HDAC inhibitor, reported previously by Scott *et al.*^73^

The DC_50_ values of the hydroxamic acid-based PROTACs ranged from 28–48 nM with corresponding *D*_max_ values of 77-83%. The TFMO-based PROTACs produced DC_50_ values ranging from 3–4 nM with associated *D*_max_ values ranging from 72–85%. Interestingly, HDAC4 degradation efficiency was not impacted by varying the PEG linker length. PROTACs 17 and 18, which are representative degraders from each series, were capable of producing a dose-dependent degradation of HDAC4 in Jurkat E6-1 cells. Additionally, PROTAC 17 exhibited the hook effect at 10 μM. By employing multiplexed western blotting, Macabuag *et al.* evaluated isoform-selectivity of PROTACs 17 and 18 for HDAC4 over other class IIa HDAC isoforms, whereby Jurkat E6-1 cells were treated with the degrader compounds over 24 h at increasing concentrations. PROTACs 17 and 18 exhibited a dose-dependent degradation of HDAC4, while no degradation of HDAC1, HDAC5, HDAC7 or HDAC9 was observed; however, minor degradation of HDAC3 was noted for PROTAC 18 at 10 μM. The study proved that PROTACs 17 and 18 induced HDAC4-selective degradation with no degradation of other class IIa isoforms. Macabuag *et al.* also demonstrated concentration-dependent degradation of HDAC4 in mouse neuroblastoma cells (Neuro-2a) over 24 h with PROTAC 18.^71^ However, a 20-fold decrease in degradation efficiency was noted in Neuro-2a cells in comparison to the Jurkat cell line. Yet, again, demonstrating how the cell type can influence the degradation efficiency of the PROTAC.

## PROTACs targeting class IIb HDACs

**Chart 5:**
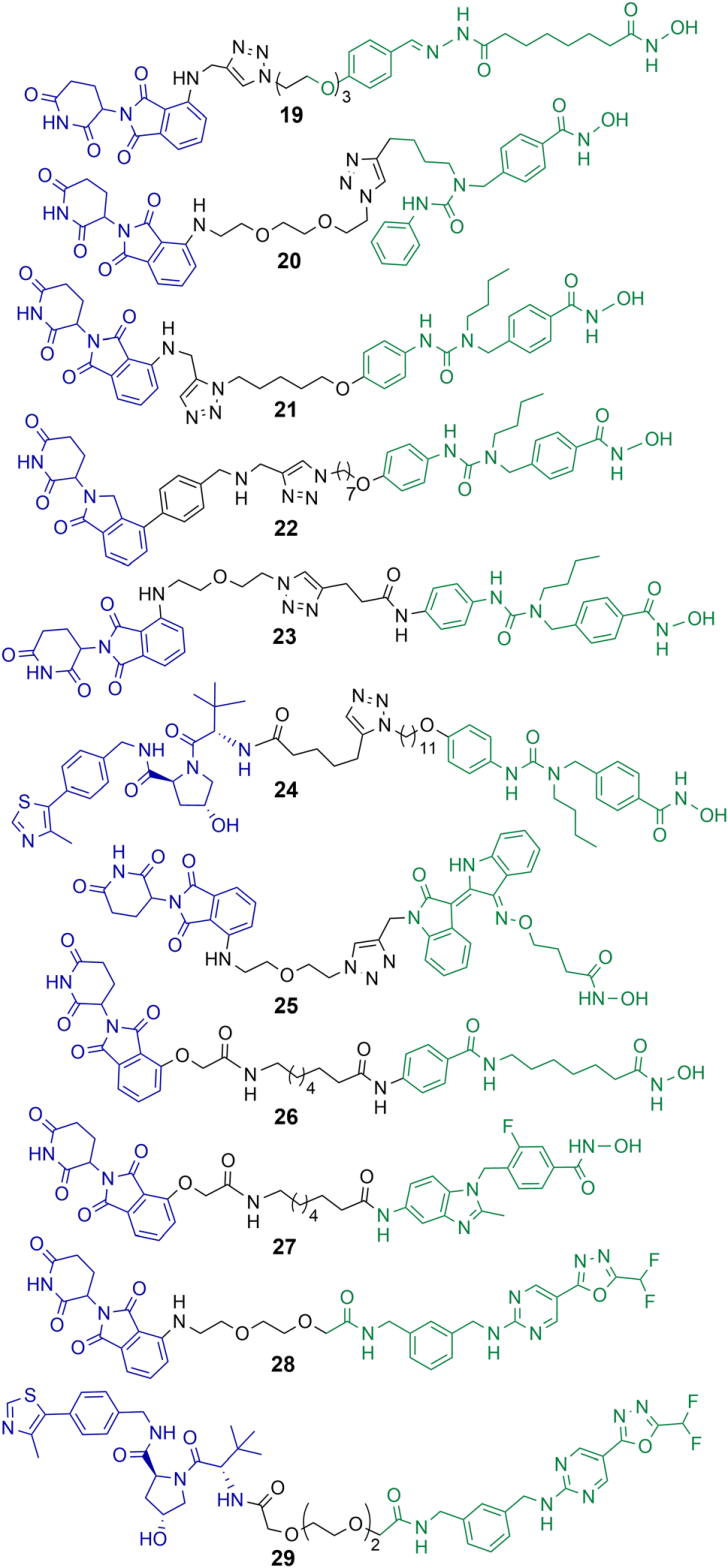
PROTACs reported for the selective degradation of HDAC6. 19 HDAC6 DC_50_ = 34 nM in MCF-7 cells;^50^20 HDAC6 DC_50_ = 3.8 nM in MM.1S cells;^76^21 HDAC6 DC_50_ = 1.6 nM in MM.1S cells and also degrades IKZF1/3;^77^22 degrades HDAC6 > 50% at 10 nM;^78^23 HDAC6 DC_50_ = 3.2 nM in MM.1S cells;^79^24 HDAC6 DC_50_ = 7.1 nM in MM.1S cells;^80^25 HDAC6 DC_50_ = 108.9 nM in K562 cells;^81^26 and 27 exhibit DC_50_ values of 3.5 nM and 19.4 nM respectively for HDAC6 in HL-60 cells.^82^28 and 29 exhibit DC_50_ values of 131 nM and 171 nM for HDAC6 respectively in MM.1S cells.^84^

Class IIb HDACs incorporate HDAC6 and HDAC10. Of the eleven-zinc dependent HDAC enzymes PROTACs targeting HDAC6 for degradation are probably the most frequently reported in the literature to date. When the first hydroxamic acid pan-zinc dependent HDAC inhibitor was incorporated into PROTAC 19 by K. Yang *et al.* it was discovered that the selective degradation of HDAC6 was observed over the other eleven zinc-dependent HDACs (Chart 5).^50^ Given this result, and the number of potent HDAC6 PROTACs reported to date in the literature, it does seem that HDAC6 is the most amenable zinc dependent HDAC isoform to proteasome mediated degradation by PROTACs. Of the HDAC isoforms HDAC6 does contain a zinc-finger ubiquitin-binding domain,^74^ and it is tempting to speculate that this ubiquitin binding domain is a potential reason why HDAC6 is so amenable to proteasome mediated degradation by PROTACs over other HDAC isoforms. In direct comparison there are single digit nanomolar inhibitors reported for HDAC10,^75^ however to date, as of writing, no PROTACs targeting HDAC10 for degradation have been reported.

## PROTACs targeting HDAC6

After K. Yang *et al.* reported the selective degradation of HDAC6 with PROTAC 19 incorporating a pan-HDAC inhibitor as the HDAC ligand,^50^ An *et al.* utilised the selective HDAC6 inhibitor Nexturastat A for inspiration in designing PROTACs to target HDAC6.^76^ PROTAC 20 incorporating Nexturastat A as the HDAC ligand and pomalidomide as the E3-ligand exhibited an impressive DC_50_ value of 3.8 nM in the B lymphoblast MM.1S. cell line (Chart 5). No degradation of HDAC1, HDAC2 or HDAC4 was observed within 24 hours in the presence of 20. In cell viability assays 20 was directly comparable to Nexturastat A, exhibiting a GI_50_ value of 1.21 μM compared to a value of 2.25 μM for Nexturastat A.

Wu *et al.* reported a potent and selective HDAC6 degrader 21 also utilising Nexturastat A as the HDAC ligand,^77^ with a different linker attachment position to the ligand to PROTAC 20. Again, PROTAC 21 exhibited a single digit nanomolar DC_50_ value of 1.6 nM in MM.1S cells and could initiate HDAC6 degradation within 1 hour of treatment. PROTAC 21 also degraded IKZF1/3 in a dose dependent manner, known neo-substrates of PROTACs incorporating thalidomide analogues to recruit the cereblon E3 ligase. Above concentrations of 100 nM this dual HDAC6 and IKZF1/3 degradation by 21 was found to be responsible for enhanced antiproliferation effects in MM.1S cells. To abolish IKZF1/3 degradation, K. Yang *et al.* were able to optimise cereblon recruiting E3-ligands and incorporate them into PROTACs such as 22 to promote the selective degradation of HDAC6 without the co-degradation of IKZFs.^78^ Highlighting that IKZF degradation does not always have to coincide with PROTACs incorporating cereblon recruiting E3-ligases. In a different study by H. Yang *et al.*, the authors were able to demonstrate that alteration of the linker connectivity to Nexturastat A in PROTAC 23 still maintained potent DC_50_ values directly comparable to other HDAC6 targeting PROTACs.^79^

K. Yang *et al.* reported PROTAC 24 recruiting the VHL E3 ligase to selectively degrade HDAC6 with a DC_50_ value of 7.1 nM and *D*_max_ value of 95% in MM.1S cells.^80^ Utilising the VHL ligand for HDAC6 degradation required a longer linker length to target HDAC6 with similar *D*_max_ values to cereblon recruiting HDAC6 PROTACs. One of the potential advantages of 24, similar to 22, is that IKZF1/3 neo-substrates were not degraded when recruiting the VHL E3 ligase and this could be considered a more selective chemical probe for studying HDAC6 related biology. No cell viability assays were reported with 24 in this study.

Cao *et al.* designed and synthesised a structurally unique HDAC6 targeting PROTAC 25,^81^ based on the natural product indirubin as the ligand to engage HDAC6. PEG linkers were explored in this study and intriguingly the shortest linker containing one PEG unit was much more effective at HDAC6 degradation than the longer PEG linkers. 25 exhibited a HDAC6 DC_50_ value of 108.9 nM in K562 lymphoblast cells with no degradation of HDAC1 observed. The authors were interested in investigating NLRP3 inflammasome activation with 25, and 25 reduced the expression of NLRP3 in THP-1 cells. The selective HDAC6 inhibitor from which PROTAC 25 was derived was less effective in this response, and the inhibitor was also more cytotoxic to cells.

Sinatra *et al.* utilised their novel solid phase synthesis approach to hydroxamic acid-based PROTACs in an attempt to discover PROTACs to selectively degrade HDAC6.^82^ They identified 26 and 27 with DC_50_ values of 3.5 nM and 19.4 nM respectively for HDAC6 in the leukaemia HL-60 cell line. No degradation of HDAC1 or HDAC4 was observed with 26 and 27. 26 and 27 were screened against a small panel of acute myeloid leukaemia cell lines and B-cell acute lymphoblastic leukaemia cell lines for their effects on cell viability. Despite its potent DC_50_ value for HDAC6, 27 did not compromise cell viability in the cell lines tested at concentrations ranging from 0.5–50 μM, the authors noted that this is an observation similar to others studying the effects of selective HDAC6 inhibitors on cell proliferation.^83^ PROTAC 26, on the other hand, did compromise cell viability in three of the AML cell lines tested and was capable of inducing apoptosis, but with IC_50_ values in the double digit micromolar ranges. This was a surprising result given that 26 is not only a selective HDAC6 degrader but also exhibits a similar inhibition profile to class I HDACs as the pan-HDAC inhibitor vorinostat *in vitro*. The authors hypothesised that the reduced permeability of 26 to the nucleus compared to vorinostat is perhaps responsible for the loss of potency in cell viability assays.

The same group reported non-hydroxamic acid based PROTACs 28 and 29 that selectively degrade HDAC6 in MM.1S cells.^84^ Offering an alternative to the hydroxamic acid functional group which has previously been reported for mutagenic effects.^23^ One of the most intriguing findings of this study was that 28 and 29 were compromised in terms of their inhibition of HDAC6 compared to hydroxamic acid HDAC inhibitors, with IC_50_ values of 0.64 μM and 0.69 μM respectively. Yet, 28 and 29 were more potent degraders of HDAC6 in cells, with DC_50_ values of 131 nM and 171 nM respectively, both PROTACs exhibited a hook effect at 10 μM. Although extensive studies were not carried out their effects on cell viability, these PROTACs did not compromise cell viability in the MM.1S cells in which they were tested.

## PROTACs targeting Class III HDACs

**Chart 6:**
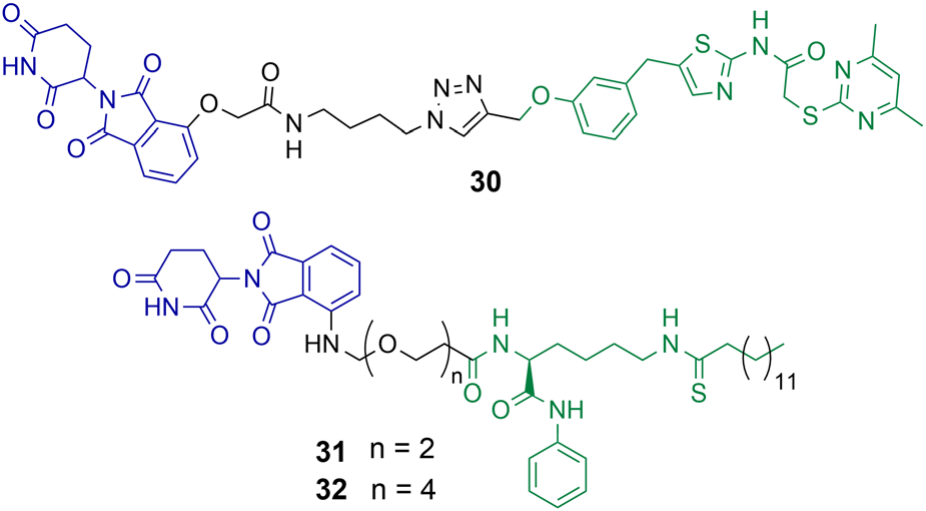
PROTACs reported for the selective degradation of SIRT2. 30 degrades SIRT2 selectively over SIRT 1 at a concertation range of 0.2–10 μM in HeLa cells.^49^31 and 32 degrade SIRT2 at concentration range of 0.5–10 μM in MCF7 and BT-549 cells with no SIRT1 degradation observed.^85^

Class III HDACs utilise NAD^+^ to catalyse the hydrolysis of acetylated lysine residues and consist of the sirtuin family SIRT1-7. The first HDAC targeting PROTAC reported in the literature was targeting SIRT2 for degradation.^49^ PROTAC 30 was synthesised utilising click chemistry and the POI ligand incorporated a selective inhibitor of SIRT2 and thalidomide as the E3-ligand (Chart 6).^49^ Dose dependent degradation of SIRT2 was observed in HeLa cells with no degradation of SIRT1. SIRT2 degradation by 30 could be further visualised in living HeLa cells with GFP-tagged SIRT2.

J.Y. Hong *et al.* reported a new SIRT2 inhibitor and incorporated it into PROTACs 31 and 32.^85^31 and 32 were capable of degrading SIRT2 at concentration range of 0.5–10 μM in MCF7 and BT-549 cell lines, with a hook effect observed at greater concentrations. *Via* degradation 32 was capable of reducing SIRT2 deacetylase and defatty acylation activity in cells, whereby the SIRT2 inhibitor from which PROTAC 32 was derived was not capable of reducing SIRT2 defatty acylation activity. This could be a major advantage to using PROTACs to degrade SIRTs over inhibition of their catalytic active site alone. At lower concentrations 32 was more effective at compromising cell viability in MCF7 and BT-549 cells than the inhibitor from which it was derived, however at higher concentrations the differences between 32 and the selective SIRT2 inhibitor were much less. The authors hypothesised this is due to the hook effect exhibited on SIRT2 in the presence of 32.

## PROTACs targeting class IV – HDAC 11

HDAC11 is the singular HDAC isoenzyme in class IV and was the most recently discovered HDAC in 2002.^86^ It is the smallest of the HDAC enzymes and is a much more effective fatty-acid deacylase than a histone deacetylase.^87^ HDAC11 does not exhibit significant effects on cell proliferation but has been noted as a potential target for metabolic disorders.^88^ Selective inhibitors of HDAC11 have been reported,^89^ however as of yet, at the time of writing, no PROTACs targeting HDAC11 for degradation have been reported. HDAC11 could potentially be a very amenable target for proteasome-mediated degradation as Long *et al.* noted its poly-ubiquitination and short half-life of 4 hours in BEAS-2B cells, which could be reversed in the presence of the proteasome inhibitor MG132.^90^

## Summary and future outlooks

It is clear that PROTACs have the capacity to offer potent and selective degradation of individual HDAC isoforms in the cell. PROTACs are already being used as chemical probes to study biology related to individual HDAC isoforms. Degrading the HDAC enzyme *via* the proteasome, rather than solely inhibiting the catalytic active site, will allow researchers to probe the biology of HDACs in cells and *in vivo* beyond their enzymatic function alone. Five years ago, before the first HDAC targeting PROTAC was reported, this would have been non-trivial, at least by small molecule approaches in chemical biology. It is also clear that the selectivity of degradation is not only PROTAC dependent but also dependent on the cell line, an observation also made by others.^91^ Before PROTACs are used as chemical probes it would be recommended to first validate the selectivity of HDAC isoform degradation in the cell line of investigation. This seems to be even more imperative when a pan-HDAC inhibitor is incorporated as the HDAC ligand in the PROTAC.

Some HDAC isoforms certainly seem more prone to PROTAC-mediated degradation than others. The proteomics study carried out by Xiong *et al.*^56^ investigating HDAC isoform degradation in the presence of 48 PROTACs correlates well with the number of isoform-selective HDAC targeting PROTACs reported to date in the literature. PROTACs capable of the selective degradation of HDAC6 are the most frequently reported, followed by selective degraders of HDAC8 and selective degraders of HDAC3. Future challenges will likely involve dialling out the degradation of these isoforms while attempting to degrade another select HDAC isoform. It will also be interesting to witness the discovery of new E3-ligands and how these new ligands can influence or modify HDAC degradation selectivity. In regards to SIRTs much fewer studies have been carried out in comparison to Zn^2+^ dependent HDACs but the selective degradation of SIRT2 over SIRT1 certainly seems to have been achieved.

In a therapeutic context, there are examples whereby HDAC targeting PROTACs are more effective at compromising cell viability in cancer cells than their counterpart HDAC inhibitor. There are also examples whereby the reverse is true, particularly when compared to a pan-HDAC inhibitor. However, in future studies, the therapeutic potential of HDAC targeting PROTACs should be investigated beyond cytotoxicity alone. For example, Banik *et al.* reported on the noncanonical and non-cytotoxic effects of selective HDAC6 inhibitors in reducing tumour growth *in vivo* by enhancing antitumor immune responses with immune checkpoint inhibitors in breast cancer.^92^ Additionally, the class I HDAC inhibitor tucidinostat was found to act synergistically with the aPD-L1 antibody to reduce solid tumours.^93^ There have been a number of studies recently reported demonstrating how HDAC inhibitors can prime and enhance the anti-tumour immune response of immune check point inhibitors and help overcome associated drug resistance to cancer immunotherapies in solid tumours.^94^ There are a significant number of active and recruiting clinical trials that are investigating combination therapies of HDAC inhibitors with immune checkpoint inhibitors.^94^ This could be another novel therapeutic application of PROTACs targeting HDACs. However, the fate of PROTACs targeting HDACs in a clinical context perhaps lies with the fate of exemplary PROTACs targeting AR and ER such as ARV-110 and ARV-471 *etc.*^46^ currently in clinical trials and their potential FDA approval status. If and when that time comes, it seems researchers will be primed and ready with a significant number of HDAC targeting PROTACs already available for optimisation to progress to clinical trials.

## Conflicts of interest

J. T. H. and J. P. S. are inventors on the PCT patent application WO2021148811A1 and the following patents: JP2023-510947, US2023-0120211A1.

## Supplementary Material
